# Ethnicity Differences in the Association of UCP1-3826A/G, UCP2-866G/A and Ala55Val, and UCP3-55C/T Polymorphisms with Type 2 Diabetes Mellitus Susceptibility: An Updated Meta-Analysis

**DOI:** 10.1155/2021/3482879

**Published:** 2021-10-19

**Authors:** Rong Huang, Tingting Cai, Yunting Zhou, Yuming Wang, Huiying Wang, Ziyang Shen, Wenqing Xia, Xiaomei Liu, Bo Ding, Yong Luo, Rengna Yan, Huiqin Li, Jindan Wu, Jianhua Ma

**Affiliations:** Department of Endocrinology, Nanjing First Hospital, Nanjing Medical University, No. 32 Gongqingtuan Road, Nanjing 210012, China

## Abstract

**Background:**

The relationship between uncoupling protein (UCP) 1-3 polymorphisms and susceptibility to type 2 diabetes mellitus (T2DM) has been extensively studied, while conclusions remain contradictory. Thus, we performed this meta-analysis to elucidate whether the UCP1-3826A/G, UCP2-866G/A, Ala55Val, and UCP3-55C/T polymorphisms are associated with T2DM.

**Methods:**

Eligible studies were searched from PubMed, Cochrane Library, and Web of Science database before 12 July 2020. Pooled odds ratios (ORs) with corresponding 95% confidence intervals (CIs) were calculated to evaluate the strength of the association. Heterogeneity analysis, subgroup analysis, sensitivity analysis, and publication bias were also performed.

**Results:**

A total of 38 case-control studies were included in this meta-analysis. The overall results revealed significant association between T2DM and the UCP2 Ala55Val polymorphism (recessive model: OR = 1.25, 95% CI 1.12-1.40, *P* < 0.01; homozygous model: OR = 1.33, 95% CI 1.03-1.72, *P* = 0.029, respectively). In subgroup analysis stratified by ethnicity, T2DM risk was increased with the UCP2 Ala55Val polymorphism (allele model: OR = 1.17, 95% CI 1.02-1.34, *P* = 0.023; recessive model: OR = 1.28, 95% CI 1.13-1.45, *P* < 0.01; homozygous model: OR = 1.39, 95% CI 1.05-1.86, *P* = 0.023, respectively), while decreased with the UCP2-866G/A polymorphism in Asians (dominant model: OR = 0.86, 95% CI 0.74-1.00, *P* = 0.045).

**Conclusions:**

Our results demonstrate that the UCP2-866G/A polymorphism is protective against T2DM, while the UCP2 Ala55Val polymorphism is susceptible to T2DM in Asians.

## 1. Introduction

Diabetes is a serious public health problem characterized by chronic hyperglycemia. The International Diabetes Federation (IDF) estimates that there were approximately 463 million adults (aged 20-79 years) diagnosed with diabetes in 2019, and this number is expected to reach 700 million by 2045 across the world [1]. Among them, type 2 diabetes mellitus (T2DM) is the most prevalent which accounts for 90%-95%. Till now, the detailed etiology of T2DM have not been fully clarified, and genetic predisposition is believed to exert great effects together with environmental influences [2].

Uncoupling proteins (UCPs) are a family of mitochondrial anion transporters located in the mitochondrial inner membrane which plays crucial roles in regulating the flux of protons through the ATP synthase [3]. There are five members described in the mammal UCP family, including UCP1 to UCP5. UCP1 is specifically expressed in the brown adipose tissue (BAT); UCP2 is more broadly expressed, including pancreatic *β* cells and cells of the immune system, skeletal muscle, spleen, liver, lung, and macrophages; UCP3 is primarily expressed in skeletal muscle, but it is also found in BAT and heart tissue; UCP4 and UCP5 are recently discovered mainly in the central nervous system [4, 5]. Previous studies have linked UCPs to energy expenditure both in animal models and in obese population, especially UCP1, UCP2, and UCP3 [6–9]. Moreover, the UCPs were also demonstrated to participate in reactive oxygen species production, oxidant stress, apoptosis, inflammation, and insulin resistance [10–14]. For those reasons, UCP1, UCP2, and UCP3 may be involved in the development of obesity, T2DM, and diabetic complications [15, 16].

Human UCP1 gene is located on chromosome 4q28-q31 and 8.9 kb in length, while both UCP2 and UCP3 genes map to chromosome 11q13 and spans 8.2 and 8.7 kb, respectively [17]. Over the past few decades, numerous studies have investigated the association between single-nucleotide polymorphisms (SNPs) of the UCP1-3 genes and T2DM susceptibility, and the most focused on the -3826A/G (rs1800592) polymorphism in the promoter region of the UCP1 gene, the -866G/A (rs659366) polymorphism in the promoter region and a missense variant in exon 4 (Ala55Val, C/T, rs660339) of the UCP2 gene, and the -55C/T (rs1800849) polymorphism in the promoter region of the UCP3 gene [18–21]. However, the results remain under debate. Consequently, this meta-analysis was carried out based on the latest publications in attempt to elucidate whether there is an association between the UCP polymorphisms and T2DM susceptibility.

## 2. Methods

This meta-analysis was performed in accordance to the Preferred Reporting Items for Systematic reviews and Meta-Analysis (PRISMA) guidelines (File S1).

### 2.1. Literature Search

We systematically searched electronic databases of PubMed, Cochrane Library, and Web of Science for all relevant articles published before 12 July 2020. The search terms were applied as follows: (“diabetes” or “T2D” or “T2DM”) and (“uncoupling protein” or “UCP”) and (“polymorphism” or “mutation” or “variant”). To obtain more qualified studies, the references cited in the original research and review articles were also manually searched. The papers were restricted to humans and written in English.

### 2.2. Literature Inclusion

Studies were considered eligible when meeting the following inclusion criteria: (1) case-control study design; (2) evaluating the association between the UCP1-3826A/G, UCP2-866G/A and Ala55Val, and UCP3-55C/T polymorphisms and T2DM susceptibility; and (3) providing sufficient genotype data to calculate odds ratios (ORs) and 95% confidence intervals (CIs). The exclusion criteria were (1) editorials, case reports, letters, comments, reviews, or meta-analyses and (2) studies without detailed genotyping data. Furthermore, if there were duplicate publications based on the same data, only the latest or most complete study was included in our meta-analysis.

### 2.3. Data Extraction

Two reviewers (Huang R and Cai TT) independently extracted the following data from the enrolled studies: first author, publication year, ethnicity, genotyping method, total number of cases and controls, genotype and allele distributions of cases and controls, and controls with Hardy-Weinberg equilibrium (HWE) or not. All possible efforts were made to contact the corresponding authors if essential data were needed. Any discrepancy in data extraction was resolved by a third reviewer (Zhou YT).

### 2.4. Quality Assessment

Two investigators (Wang YM and Wang HY) separately performed the quality assessment of each included study using the Newcastle-Ottawa quality assessment scale (NOS). The NOS comprises the following three aspects: selection of study subjects (4 points), comparability of study subjects (2 points), and exposure or outcomes (3 points) [22]. The total score ranges from 0 to 9, and those with score ≥ 6 were considered as high-quality studies.

### 2.5. Statistical Analysis

HWE of the genotype distribution in the control subjects was assessed by *χ*^2^ test. Pooled ORs with corresponding 95% CIs were used to measure the strength of the association between UCP1-3826A/G, UCP2-866G/A and Ala55Val, and UCP3-55C/T polymorphisms and T2DM susceptibility under the following models: allele model, dominant model, recessive model, homozygous model, and heterozygous model. Subgroup analysis was performed according to the ethnicity of included populations. The heterogeneity across studies was estimated via *Q* test and *I*^2^ statistics. *I*^2^ > 50% or *P*_*Q*_ ≤ 0.1 was considered to indicate significant heterogeneity. If significant heterogeneity existed, random effects model (REM) was used; otherwise, fixed effects model (FEM) was applied. Galbraith plot was conducted to explore the outlier and main contributor to heterogeneity. To assess the stability of the results, sensitivity analysis was carried out by omitting each study in sequence. Additionally, potential publication bias was evaluated with Begg's funnel plot and Egger's test. All statistical analyses were performed using STATA Version 11.0 (College Station, TX, USA), and a two-sided *P* value < 0.05 was considered statistically significant.

## 3. Results

### 3.1. Characteristics of Included Studies

**Figure 1:**
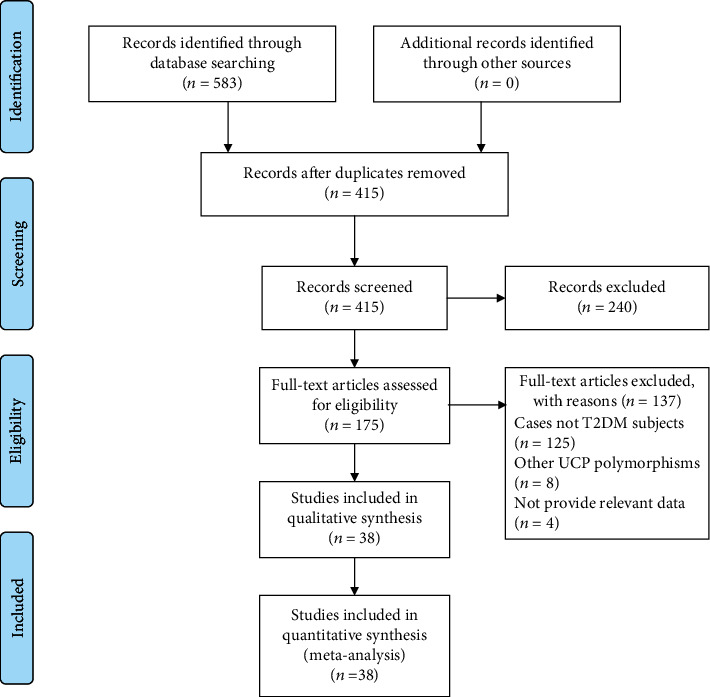
Flow chart of literature search.

As described in the flow chart, a total of 583 studies were retrieved through searching the electronic database (Figure 1). After excluding duplicated publications, 415 records were initially identified. Then, 240 articles were removed including editorials, case reports, letters, comments, reviews, and meta-analyses, and 175 articles were assessed in full. Finally, 38 relevant studies with sufficient data were included in our meta-analysis [17, 19, 20, 23–57]. Among the eligible studies, 9 analyzed the UCP1-3826A/G polymorphism, 23 analyzed the UCP2-866G/A polymorphism, 9 analyzed the UCP2 Ala55Val polymorphism, and 11 analyzed the UCP3-55C/T polymorphism.  Table 1 detailly shows the main characteristics of the studies.

### 3.2. Synthesis Analysis

**Figure 2:**
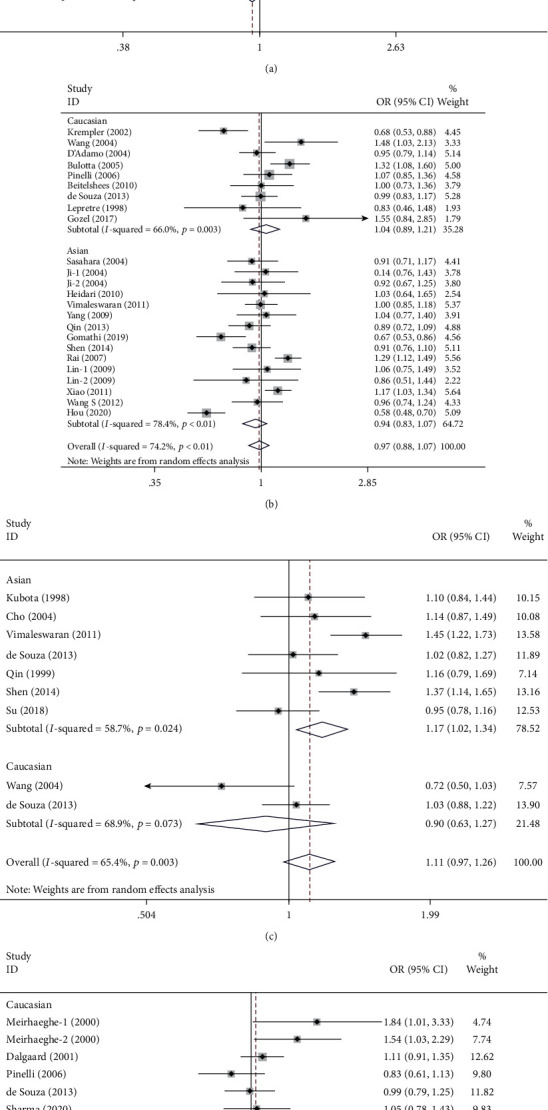
Meta-analysis for the association between the UCP polymorphisms and T2DM susceptibility stratified by ethnicity (allele model). (a) UCP1-3826A/G polymorphism; (b) UCP2-866G/A polymorphism; (c) UCP2 Ala55Val polymorphism; (d) UCP3-55C/T polymorphism. The area of the squares reflects the study-specific weight, and the diamond illustrates the summary random effects OR (95% CI).

The results of meta-analysis and heterogeneity test for the association of UCP1-3826A/G, UCP2-866G/A and Ala55Val, and UCP3-55C/T polymorphisms with T2DM susceptibility under five inheritance models are summarized in details in Table 2.  Figure 2 illustrates the pooled ORs (95% CI) of UCP1-3826A/G, UCP2-866G/A and Ala55Val, and UCP3-55C/T polymorphisms with T2DM risk stratified by ethnicity under an allele contrast inheritance model. Our results revealed significant association between T2DM and UCP2 Ala55Val polymorphism (recessive model: OR = 1.25, 95% CI 1.12-1.40, *P* < 0.01; homozygous model: OR = 1.33, 95% CI 1.03-1.72, *P* = 0.029, respectively), but no associations between T2DM and UCP1-3826A/G, UCP2-866G/A or UCP3-55C/T polymorphisms in the overall population. Further in the subgroup analyses stratified by ethnicity, T2DM risk was increased with UCP2 Ala55Val polymorphism (allele model: OR = 1.17, 95% CI 1.02-1.34, *P* = 0.023; recessive model: OR = 1.28, 95% CI 1.13-1.45, *P* < 0.01; homozygous model: OR = 1.39, 95% CI 1.05-1.86, *P* = 0.023, respectively), while decreased with UCP2-866G/A polymorphism in Asians (dominant model: OR = 0.86, 95% CI 0.74-1.00, *P* = 0.045) (Table 2).

### 3.3. Heterogeneity Analysis

As shown in Table 2, significant heterogeneity was found among studies in almost all genetic models of the overall population except the heterozygous model of the UCP2-866G/A polymorphism, the recessive model of the UCP2 Ala55Val polymorphism, and the dominant and heterozygous models of the UCP3 -55C/T polymorphism, but no heterogeneity was found in all genetic models for the UCP1-55C/T polymorphism. After stratification by ethnicity, the heterogeneity was only eliminated between the studies of the UCP3-55C/T polymorphism in populations of European descent in the recessive and homozygous genetic models, but not in Asian descent. The heterogeneity was also existed in studies of the UCP2-866G/A and Ala55Val polymorphisms both in Asian descent and European descent. Therefore, Galbraith plot analysis was performed to detect the outlier and main contributor to heterogeneity, and the results indicated that Bulotta et al. 2005 and Hou et al. 2020, Vimaleswaran et al. 2011, and Wang LL et al. 2012 were the outliers and main contributor to heterogeneity of the UCP2-866G/A, Ala55Val, and UCP-55C/T polymorphisms, respectively (Figure S1-S3).

### 3.4. Sensitivity Analysis

**Figure 3:**
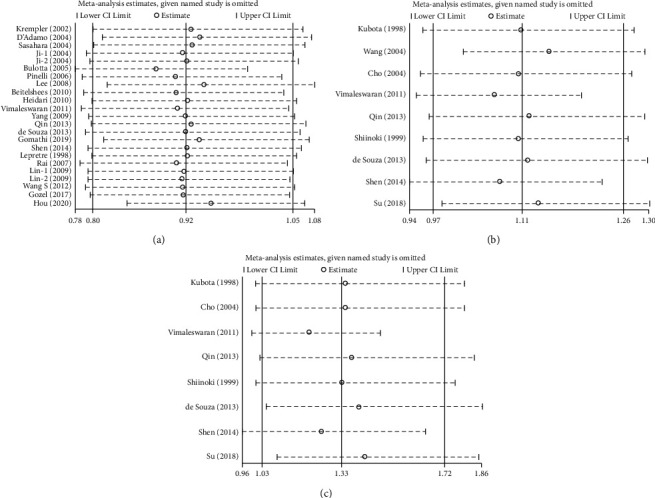
Sensitivity analysis for the association between the UCP polymorphisms and T2DM susceptibility. (a) Dominant model of the UCP2-866G/A polymorphism; (b) allele model of the UCP2 Ala55Val polymorphism; (c) homozygous model of the UCP2 Ala55Val polymorphism.

To evaluate the influence of a single study on the pooled results, sensitivity analysis was performed by sequentially omitting one study at a time in the overall population. The results showed that the pooled ORs lay within the overall range of 95% CIs after omitting any single study in all compared inheritance models, except for excluding the study of the Bulotta et al. 2005 in the dominant model of the UCP2-866G/A polymorphism, the Wang et al. 2004 in the allele model, and the Vimaleswaran et al. 2011 and the Shen et al. 2014 in the homozygous model of the UCP2 Ala55Val polymorphism (OR = 0.88, 95% CI 0.78-0.99, *P* = 0.039; OR = 1.15, 95% CI 1.02-1.29, *P* = 0.024; OR = 1.21, 95% CI 0.99-1.47, *P* = 0.064; OR = 1.25, 95% CI 0.96-1.64, *P* = 0.102, respectively) (Figure 3).

### 3.5. Publication Bias

**Figure 4:**
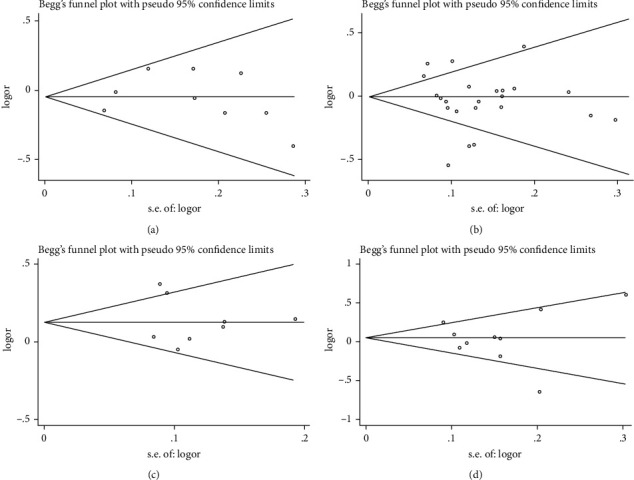
Funnel plot for the association between the UCP polymorphisms and T2DM susceptibility (allele model). (a) UCP1-3826A/G polymorphism (*P* = 0.822); (b) UCP2-866G/A polymorphism (*P* = 0.534); (c) UCP2 Ala55Val polymorphism (*P* = 0.267); (d) UCP3-55C/T polymorphism (*P* = 0.757).

Begg's funnel plot and Egger's test were conducted to assess the publication bias of the literature. As expected, the funnel plots were visually symmetrical, and all *P* values obtained from Egger's test were >0.05, which interpreted that there is no publication bias for any of the UCP polymorphisms analyzed (for example, in the allele model, Figure 4).

## 4. Discussion

T2DM is one of the most common noncommunicable diseases which is thought to be the result of interactions between complex gene-gene and gene-environment. A number of studies have examined the associations of the UCP1-3826A/G, the UCP2-866G/A, Ala55Val, and UCP3-55C/T polymorphisms with T2DM, but the results are still inconsistent. As a single study might lack sufficient power, especially when the sample size is not adequate, we designed this meta-analysis of 38 published studies from different populations to obtain a more precise conclusion. Our results showed that only the UCP2 Ala55Val polymorphism is associated with T2DM in the overall population. In a stratified analysis according to ethnicity, we found that the UCP2 Ala55Val polymorphism is significantly associated with increased risk of T2DM, while the UCP2-866G/A polymorphism is associated with decreased risk of T2DM in Asian population. However, the correlation of UCP1-3826A/G and UCP3-55C/T polymorphisms with T2DM lacked corresponding evidence in either subjects of Asian or of Caucasian descent.

The -3826A/G polymorphism in the promoter region of the UCP1 gene was found to be linked to reduced mRNA expression, which indicated that the polymorphism may be of functional importance [58]. Thus, numerous studies have been carried out to evaluate the association between this polymorphism and obesity or obesity-related disorders. Results concluded from previous meta-analyses showed that the UCP1-3826A/G polymorphism is not associated with any change in BMI or obesity regardless of the inheritance model or stratification analysis by ethnicity [59, 60]. In our study, we confirmed no relationship between the UCP1-3826A/G polymorphism and susceptibility to T2DM either in Asian population or in Caucasian population, which was also supported by a previous meta-analysis by de Souza et al. 2013 [19].

The -866G/A polymorphism in the core promoter of the UCP2 gene seems to be connected with putative binding sites for specific transcription factors [61]. Previous study revealed that the A allele of the UCP2-866G/A polymorphism contributes to insulin resistance and obesity when compared with G allele [34]. Thus, it is reasonable to draw the conclusion that the UCP2 rs659366 is significantly associated with increased risk of T2DM by Xu et al. 2021, especially in Asian population [21]. Nevertheless, there were no association found in the meta-analyses performed by Xu et al. 2011, Qin et al. 2013, and de Souza et al. 2013 [18–20]. Contradictory to all aforementioned meta-analyses, an important finding is shown in our meta-analysis that the UCP2-866G/A polymorphism is associated with decreased risk of T2DM in the dominant model in Asian population. One possible explanation for this discrepancy is that there exist conflicting data in human tissues which reported both increased and decreased UCP2 mRNA levels being associated with the -866A allele [55, 62]. For that reason, the association of the UCP2-866A allele with decreased risk of T2DM in Asians seems to be biologically receivable since an increased UCP2 mRNA expression in adipocytes would be relevant to increased energy expenditure.

The UCP2 Ala55Val variant is located in exon 4 of the UCP2 gene where the base change can lead to a conservative amino acid change from alanine (Ala) to valine (Val) [63]. Although this alteration is not predicted to cause a functional change in the corresponding protein, our results are consistent with two previous meta-analyses which find significant association between the UCP2 Ala55Val polymorphism and increased risk of T2DM, mainly in Asians [18, 19]. Nevertheless, there were no evidence of this association found in neither the Chinese population nor the whole subjects by Qin et al. 2013 and Xu et al. 2021 [20, 21]. The ethnic discrepancy in susceptibility to T2DM may be partially attributed to different distribution of genotype frequencies and lifestyle between Asian and Caucasian populations. For example, different nutrient intakes were found to influence the roles of genetic polymorphisms in obesity and obesity-related diseases [64, 65]. Thus, it is reasonable that there exists ethnicity difference in the association of UCP2 Ala55Val polymorphism with T2DM susceptibility owing to different diet patterns.

The UCP3 -55C/T promoter variant is of interest because of its position at 4 bp downstream of a peroxisome proliferator-activated receptor (PPAR) responsive region, which could modify PPAR-dependent responsiveness [66]. Thus, many studies have linked this polymorphism to the regulation of lipid metabolism and insulin sensitivity [67, 68]. Previous meta-analyses also showed that the UCP3-55C/T polymorphism is related to prominent increase in BMI, as well as risk for T2DM in Asians [18, 19, 59]. In contract, our results failed to find any association of UCP3-55C/T polymorphism with T2DM. We could not fully exclude the possibility that the latest publications included in our meta-analysis might vary the final results.

Although some previous meta-analyses reported the role of UCP polymorphisms in the risk for T2DM, our meta-analysis included the most recent publications and conducted a series of analyses, including subgroup analysis, heterogeneity analysis, sensitivity analysis, and publication bias, to achieve more accurate results. Certainly, some limitations should be acknowledged in the present study for better interpreting the results [69]. Firstly, there was substantial heterogeneity among included studies, despite the use of random effects model, which may affect the precision of the results. Secondly, sensitivity analysis of this meta-analysis indicated that the overall results were somewhat unstable. Thirdly, the small number and sample size of studies may confound the pooled results to a certain degree, especially for Caucasian origin included in the UCP2 Ala55Val polymorphism. Fourthly, due to lack of original information for each included subjects, the overall results of our study were based on individual unadjusted OR. Additionally, we only considered the role of individual polymorphism and did not take into account their interaction with other polymorphisms and environmental factors.

In conclusion, our results demonstrated that the -866G/A polymorphism is protective against T2DM, while the Ala55Val polymorphism of UCP2 gene is susceptible to T2DM in Asians. Nevertheless, given the presence of between-study heterogeneity and confounding factors in this meta-analysis, further well-designed and large-scale studies, particularly, studies that take the effects of gene-gene and gene-environment interactions into consideration, should be conducted to verify the current findings.

## Figures and Tables

**Table 1:** Characteristics of UCP1-3826A/G, UCP2-866G/A and Ala55Val, and UCP3-55C/T polymorphisms from included studies in the meta-analysis.

First author	Year	Ethnicity	Genotyping method		Case					Control						Controls with HWE	Score
				Total	ww	mw	mm	w	m	Total	ww	mw	mm	w	m		
UCP1-3826A/G																	
Boullu-Sanchis	1999	Asian	PCR-RFLP	89	30	13	46	73	105	100	38	14	48	90	110	No	7
Heilbronn	2000	Caucasian	PCR-RFLP	45	22	19	4	63	27	99	59	36	4	154	44	Yes	6
Sivenius	2000	Caucasian	PCR-RFLP	70	38	20	12	96	44	123	65	32	26	162	84	Yes	9
Mori	2001	Asian	PCR-RFLP	320	83	156	81	322	318	250	58	116	76	232	268	Yes	7
Lindholm	2004	Caucasian	PCR-RFLP	434	253	181		ND	ND	106	68	38		ND	ND	Yes	7
Sramkova	2007	Caucasian	PCR-RFLP	295	157	124	14	438	152	120	61	49	10	171	69	Yes	7
Lin-1	2009	Asian	TaqMan	178	42	79	57	163	193	108	24	54	30	102	114	Yes	9
Lin-2	2009	Asian	TaqMan	184	44	91	49	179	189	37	12	15	10	39	35	Yes	9
Vimaleswaran	2010	Asian	PCR-RFLP	810	292	372	146	956	664	990	396	446	148	1238	742	Yes	8
de Souza	2013	Caucasian	TaqMan	981	489	370	122	1348	614	534	263	211	60	737	331	Yes	8
UCP2-866G/A																	
Lepretre	1998	Caucasian	PCR-RFLP	49	4	25	20	33	65	50	7	24	19	38	62	Yes	8
Krempler	2002	Caucasian	PCR-RFLP	201	65	106	30	236	166	391	186	156	49	528	254	Yes	9
D'Adamo	2004	Caucasian	PCR-RFLP	483	222	197	64	641	325	559	247	260	52	754	364	Yes	8
Ji-1	2004	Asian	PCR-RFLP	184	53	94	37	200	168	134	37	69	28	143	125	Yes	7
Ji-2	2004	Asian	PCR-RFLP	158	35	79	44	149	167	156	39	76	41	154	158	Yes	7
Sasahara	2004	Asian	PCR-RFLP	413	116	205	92	437	389	172	50	90	32	190	154	Yes	7
Wang	2004	Caucasian	Pyrosequencing	131	ND	ND	ND	176	86	118	ND	ND	ND	137	99	Yes	7
Bulotta	2005	Caucasian	PCR-RFLP	746	374	317	55	1065	427	327	142	144	41	428	226	Yes	8
Pinelli	2006	Caucasian	ASA	342	167	145	30	479	205	305	147	124	34	418	192	Yes	8
Rai	2007	Asian	PCR-RFLP	762	320	351	91	991	533	924	286	518	120	1090	758	No	6
Lee	2008	Asian	TaqMan	753	529		224	ND	ND	630	488		142	ND	ND	Yes	6
Lin-1	2009	Asian	TaqMan	178	59	90	29	208	148	107	33	56	18	122	92	Yes	9
Lin-2	2009	Asian	TaqMan	184	73	88	23	234	134	38	19	13	6	51	25	Yes	9
Yang	2009	Asian	PCR-RFLP	199	56	124	19	236	162	155	41	99	15	181	129	No	6
Beitelshees	2010	Caucasian	Pyrosequencing or TaqMan	107	37	56	14	130	84	341	132	151	58	415	267	No	6
Heidari	2010	Asian	PCR-RFLP	75	29	38	8	96	54	75	27	41	7	95	55	Yes	8
Vimaleswaran	2011	Asian	PCR-RFLP	487	185	239	63	609	365	919	358	432	129	1148	690	Yes	8
Xiao	2011	Asian	PCR-RFLP	930	ND	ND	ND	986	874	867	ND	ND	ND	850	884	Yes	7
Wang S	2012	Asian	PCR-RFLP	370	113	169	88	395	345	166	55	71	40	181	151	Yes	8
de Souza	2013	Caucasian	TaqMan	778	272	372	134	916	640	435	152	211	72	515	355	Yes	8
Qin	2013	Asian	PCR-RFLP	354	88	184	82	360	348	363	102	187	74	391	335	Yes	6
Shen	2014	Asian	DNA sequencing	454	140	217	97	497	411	448	153	205	90	511	385	Yes	8
Gozel	2017	Caucasian	PCR-RFLP	50	26	23	1	75	25	50	19	28	3	66	34	Yes	8
Gomathi	2019	Asian	PCR-RFLP	318	128	147	43	403	233	312	164	121	27	449	175	Yes	7
Hou	2020	Asian	PCR-RFLP	470	174	225	71	573	367	536	284	214	38	782	290	Yes	7
UCP2 Ala55Val																	
Kubota	1998	Asian	PCR-RFLP	210	60	107	43	227	193	218	64	97	57	225	211	Yes	6
Shiinoki	1999	Asian	PCR-RFLP	100	30	53	17	113	87	120	28	71	21	127	113	No	6
Cho	2004	Asian	PCR-RFLP	500	158	227	115	543	457	133	30	76	27	136	130	Yes	7
Wang	2004	Caucasian	Pyrosequencing	131	ND	ND	ND	97	165	118	ND	ND	ND	106	130	Yes	7
Vimaleswaran	2011	Asian	PCR-RFLP	487	264	198	25	726	248	919	408	412	99	1228	610	Yes	8
de Souza	2013	Caucasian	TaqMan	784	265	371	148	901	667	453	142	229	82	513	393	Yes	8
Qin	2013	Asian	PCR-RFLP	292	55	147	90	257	327	369	59	203	107	321	417	Yes	6
Shen	2014	Asian	DNA sequencing	472	166	219	87	551	393	441	121	204	116	446	436	Yes	8
Su	2018	Asian	MALDI-TOF-MS	387	132	191	64	455	319	398	142	194	62	478	318	Yes	7
UCP3-55C/T																	
Meirhaeghe-1	2000	Caucasian	NA	49	36	13	0	85	13	894	542	312	40	1396	392	Yes	8
Meirhaeghe-2	2000	Caucasian	NA	171	116	49	6	281	61	124	70	46	8	186	62	Yes	8
Dalgaard	2001	Caucasian	NA	455	253	169	33	675	235	521	280	192	49	752	290	Yes	7
Cho	2004	Asian	PCR-RFLP	499	251	204	44	706	292	132	62	59	11	183	81	Yes	7
Lindholm	2004	Caucasian	PCR-RFLP	434	220	214		ND	ND	106	51	55		ND	ND	Yes	7
Pinelli	2006	Caucasian	ASA	342	240	94	8	574	110	305	224	78	3	526	84	Yes	8
Lee	2008	Asian	TaqMan	753	381	372		ND	ND	630	296	334		ND	ND	Yes	6
Vimaleswaran	2011	Asian	PCR-RFLP	487	278	180	29	736	238	919	460	377	82	1297	541	Yes	8
Wang LL	2012	Asian	PCR-RFLP	100	41	25	34	107	93	113	67	21	25	155	71	No	7
de Souza	2013	Caucasian	TaqMan	822	559	231	32	1349	295	351	239	99	13	577	125	Yes	8
Su	2018	Asian	MALDI-TOF-MS	394	180	182	32	542	246	398	192	175	31	559	237	Yes	7
Sharma	2020	Caucasian	TaqMan	425	ND	ND	ND	748	102	342	ND	ND	ND	598	86	Yes	7

UCP: uncoupling protein; T2DM: type 2 diabetes mellitus; HWE: Hardy-Weinberg equilibrium; PCR-RFLP: polymerase chain reaction-restriction fragment length polymorphism; ASA: allele specific amplification; MALDI-TOF-MS: matrix-assisted laser desorption/ionization time of flight mass spectrometry; ND: no data. For each SNPs, w: wild allele; m: mutation allele; ww: wild homozygote; mw: mutation heterozygote; mm: mutation homozygote.

**Table 2:** Meta-analysis and heterogeneity test of UCP1-3826A/G, UCP2-866G/A and Ala55Val, and UCP3-55C/T polymorphisms with T2DM susceptibility.

Inheritance model	Overall					Caucasian					Asian				
	*n*	*I* ^2^ (%)	*P*_*Q*_	OR (95% CI)	*P*	*n*	*I* ^2^ (%)	*P*_*Q*_	OR (95% CI)	*P*	*n*	*I* ^2^ (%)	*P*_*Q*_	OR (95% CI)	*P*
UCP1-3826A/G															
Allele	9	13.0	0.326	0.95 (0.88-1.03)	0.242	4	3.4	0.376	1.00 (0.88-1.15)	0.966	5	22.6	0.271	0.92 (0.83-1.02)	0.130
Dominant	9	12.4	0.332	0.93 (0.80-1.08)	0.367	4	28.5	0.241	0.98 (0.75-1.30)	0.909	5	15.3	0.317	0.91 (0.76-1.09)	0.318
Recessive	10	0.0	0.679	0.93 (0.83-1.04)	0.230	5	0.0	0.585	0.98 (0.83-1.15)	0.769	5	0.0	0.527	0.90 (0.77-1.05)	0.167
Homozygous	9	17.0	0.291	0.91 (0.77-1.07)	0.251	4	29.3	0.236	1.00 (0.75-1.33)	0.980	5	16.1	0.312	0.87 (0.71-1.06)	0.165
Heterozygous	9	0.0	0.517	0.95 (0.81-1.12)	0.559	4	15.6	0.314	0.97 (0.72-1.31)	0.831	5	0.0	0.461	0.95 (0.78-1.15)	0.576

UCP2-866G/A															
Allele	24	74.2	<0.001	0.97 (0.88-1.07)	0.595	9	66.0	0.003	1.04 (0.89-1.21)	0.630	15	78.4	<0.001	0.94 (0.83-1.07)	0.337
Dominant	23	47.4	0.006	0.92 (0.80-1.05)	0.208	8	53.1	0.037	1.06 (0.81-1.40)	0.651	15	40.6	0.052	*0.86 (0.74-1.00)*	*0.045*
Recessive	22	74.0	<0.001	0.93 (0.80-1.07)	0.307	8	65.5	0.005	0.97 (0.78-1.21)	0.782	14	78.2	<0.001	0.90 (0.80-1.07)	0.327
Homozygous	22	63.3	<0.001	0.90 (0.75-1.09)	0.298	8	62.0	0.010	1.02 (0.73-1.43)	0.909	14	65.1	<0.001	0.85 (0.67-1.08)	0.179
Heterozygous	22	13.5	0.280	0.96 (0.87-1.06)	0.410	8	47.1	0.067	1.05 (0.88-1.26)	0.587	14	0.0	0.727	0.92 (0.81-1.04)	0.169

UCP2 Ala55Val															
Allele	9	65.4	0.003	1.11 (0.97-1.28)	0.126	2	68.9	0.073	0.90 (0.63-1.27)	0.534	7	58.7	0.024	*1.17 (1.02-1.34)*	*0.023*
Dominant	8	62.9	0.009	1.17 (0.92-1.47)	0.196	1	—	—	0.95 (0.70-1.28)	0.735	7	64.4	0.010	1.21 (0.93-1.58)	0.161
Recessive	8	33.7	0.159	*1.25 (1.12-1.40)*	*<0.01*	1	—	—	1.12 (0.87-1.43)	0.376	7	37.5	0.143	*1.28 (1.13-1.45)*	*<0.01*
Homozygous	8	58.2	0.019	*1.33 (1.03-1.72)*	*0.029*	1	—	—	1.03 (0.74-1.45)	0.846	7	57.6	0.028	*1.39 (1.05-1.86)*	*0.023*
Heterozygous	8	56.7	0.024	1.09 (0.86-1.36)	0.481	1	—	—	0.90 (0.65-1.23)	0.503	7	58.9	0.024	1.12 (0.87-1.46)	0.380

UCP3-55C/T															
Allele	10	67.3	0.001	1.04 (0.90-1.22)	0.582	6	46.8	0.094	1.10 (0.93-1.30)	0.274	4	83.4	<0.001	0.94 (0.69-1.26)	0.693
Dominant	9	36.8	0.124	1.10 (0.89-1.35)	0.381	5	14.8	0.320	1.20 (0.86-1.67)	0.281	4	60.2	0.057	1.04 (0.80-1.35)	0.790
Recessive	11	53.1	0.019	1.07 (0.93-1.24)	0.329	6	32.5	0.192	1.10 (0.92-1.32)	0.290	5	71.2	0.008	1.02 (0.79-1.30)	0.905
Homozygous	9	54.1	0.026	1.05 (0.74-1.49)	0.792	5	28.1	0.234	1.19 (0.74-1.91)	0.469	4	73.7	0.010	0.94 (0.54-1.65)	0.834
Heterozygous	9	0.0	0.782	1.11 (0.89-1.39)	0.360	5	0.0	0.541	1.14 (0.81-1.63)	0.451	4	0.0	0.655	1.09 (0.82-1.45)	0.571

UCP: uncoupling protein; T2DM: type 2 diabetes mellitus; *P*_*Q*_: *P* value for *Q* test; OR: odds ratio; CI: confidence interval.

## Data Availability

The data used to support the findings of this study are available from the corresponding author upon request.

## Supplementary Materials

Supplementary Materials File S1: PRISMA 2009 Checklist. Figure S1: Galbraith plot of UCP2-866G/A polymorphism and T2DM risk. (a) Allele model; (b) dominant model; (c) recessive model; (d) homozygous model. Figure S2: Galbraith plot of UCP2 Als55Val polymorphism and T2DM risk. (a) Allele model; (b) dominant model; (c) homozygous model; (d) heterozygous model. Figure S3: Galbraith plot of UCP3-55C/T polymorphism and T2DM risk. (a) Allele model; (b) recessive model; (c) homozygous model.

## References

[1] Saeedi P., Petersohn I., Salpea P. (2019). Global and regional diabetes prevalence estimates for 2019 and projections for 2030 and 2045: Results from the International Diabetes Federation Diabetes Atlas, 9^th^ edition. *Diabetes research and clinical practice*.

[2] Tremblay J., Hamet P. (2019). Environmental and genetic contributions to diabetes. *Metabolism: clinical and experimental*.

[3] Busiello R. A., Savarese S., Lombardi A. (2015). Mitochondrial uncoupling proteins and energy metabolism. *Frontiers in physiology*.

[4] Giralt M., Villarroya F. (2017). Mitochondrial uncoupling and the regulation of glucose homeostasis. *Current Diabetes Reviews*.

[5] Ramsden D. B., Ho P. W., Ho J. W. (2012). Human neuronal uncoupling proteins 4 and 5 (UCP4 and UCP5): structural properties, regulation, and physiological role in protection against oxidative stress and mitochondrial dysfunction. *Brain and behavior*.

[6] Gonzalez-Muniesa P., Milagro F. I., Campion J., Martinez J. A. (2006). Reduction in energy efficiency induced by expression of the uncoupling protein, UCP1, in mouse liver mitochondria. *International journal of molecular medicine*.

[7] Ishizawa M., Mizushige K., Noma T. (2006). An antioxidant treatment potentially protects myocardial energy metabolism by regulating uncoupling protein 2 expression in a chronic *β*-adrenergic stimulation rat model. *Life Sciences*.

[8] Lombardi A., Busiello R. A., de Matteis R. (2019). Absence of uncoupling protein-3 at thermoneutrality impacts lipid handling and energy homeostasis in mice. *Cells*.

[9] Taghadomi Masoumi Z., Eshraghian M. R., Hedayati M., Pishva H. (2018). Association between uncoupling protein 2, adiponectin and resting energy expenditure in obese women with normal and low resting energy expenditure. *Gynecological Endocrinology*.

[10] Jastroch M. (2017). Uncoupling protein 1 controls reactive oxygen species in brown adipose tissue. *Proceedings of the National Academy of Sciences of the United States of America*.

[11] Robson-Doucette C. A., Sultan S., Allister E. M. (2011). *β*-Cell uncoupling protein 2 regulates reactive oxygen species production, which influences both insulin and glucagon secretion. *Diabetes*.

[12] Cao T., Dong Y., Tang R., Chen J., Zhang C. Y., Zen K. (2013). Mitochondrial uncoupling protein 2 protects splenocytes from oxidative stress- induced apoptosis during pathogen activation. *Cellular immunology*.

[13] Demine S., Renard P., Arnould T. (2019). Mitochondrial uncoupling: a key controller of biological processes in physiology and diseases. *Cells*.

[14] van Dierendonck X., Sancerni T., Alves-Guerra M. C., Stienstra R. (2020). The role of uncoupling protein 2 in macrophages and its impact on obesity- induced adipose tissue inflammation and insulin resistance. *The Journal of biological chemistry*.

[15] Zhang C. Y., Baffy G., Perret P. (2001). Uncoupling Protein-2 Negatively Regulates Insulin Secretion and Is a Major Link between Obesity, *β* Cell Dysfunction, and Type 2 Diabetes. *Cell*.

[16] Dalgaard L. T., Pedersen O. (2001). Uncoupling proteins: functional characteristics and role in the pathogenesis of obesity and type II diabetes. *Diabetologia*.

[17] Lee H. J., Ryu H. J., Shin H. D. (2008). Associations between polymorphisms in the mitochondrial uncoupling proteins (_UCPs_) with T2DM. *Clinica Chimica Acta*.

[18] Xu K., Zhang M., Cui D. (2011). UCP2-866G/A and Ala55Val, and UCP3-55C/T polymorphisms in association with type 2 diabetes susceptibility: a meta-analysis study. *Diabetologia*.

[19] de Souza B. M., Brondani L. A., Bouças A. P. (2013). Associations between UCP1-3826A/G, UCP2-866G/A, Ala55Val and Ins/Del, and UCP3-55C/T polymorphisms and susceptibility to type 2 diabetes mellitus: case-control study and meta-analysis. *PLoS One*.

[20] Qin L. J., Wen J., Qu Y. L., Huang Q. Y. (2013). Lack of association of functional UCP2-866G/A and Ala55Val polymorphisms and type 2 diabetes in the Chinese population based on a case-control study and a meta-analysis. *Genetics and Molecular Research*.

[21] Xu L., Chen S., Zhan L. (2021). Association of uncoupling protein-2 -866G/A and Ala55Val polymorphisms with susceptibility to type 2 diabetes mellitus. *Medicine*.

[22] Cook D. A., Reed D. A. (2015). Appraising the quality of medical education research Methods. *Academic medicine: journal of the Association of American Medical Colleges*.

[23] Kubota T., Mori H., Tamori Y. (1998). Molecular screening of uncoupling protein 2 gene in patients with noninsulin-dependent diabetes mellitus or obesity. *Journal of Clinical Endocrinology & Metabolism*.

[24] Lepretre F., Vionnet N., Budhan S. (1998). Genetic studies of polymorphisms in ten non-insulin-dependent diabetes mellitus candidate genes in Tamil Indians from Pondichery. *Diabetes & metabolism*.

[25] Boullu-Sanchis S., Lepretre F., Hedelin G. (1999). Type 2 diabetes mellitus: association study of five candidate genes in an Indian population of Guadeloupe, genetic contribution of FABP2 polymorphism. *Diabetes & metabolism*.

[26] Shiinoki T., Suehiro T., Ikeda Y. (1999). Screening for variants of the uncoupling protein 2 gene in Japanese patients with non--insulin-dependent diabetes mellitus. *Metabolism: clinical and experimental*.

[27] Heilbronn L. K., Kind K. L., Pancewicz E., Morris A. M., Noakes M., Clifton P. M. (2000). Association of -3826 G variant in uncoupling protein-1 with increased BMI in overweight Australian women. *Diabetologia*.

[28] Meirhaeghe A., Amouyel P., Helbecque N. (2000). An uncoupling protein 3 gene polymorphism associated with a lower risk of developing type II diabetes and with atherogenic lipid profile in a French cohort. *Diabetologia*.

[29] Sivenius K., Valve R., Lindi V., Niskanen L., Laakso M., Uusitupa M. (2000). Synergistic effect of polymorphisms in uncoupling protein 1 and *β*_3_-adrenergic receptor genes on long-term body weight change in Finnish type 2 diabetic and non-diabetic control subjects. *International journal of obesity and related metabolic disorders: journal of the International Association for the Study of Obesity*.

[30] Dalgaard L. T., Hansen T., Urhammer S. A., Drivsholm T., Borch-Johnsen K., Pedersen O. (2001). The uncoupling protein 3-55 C -->T variant is not associated with type II diabetes mellitus in Danish subjects. *Diabetologia*.

[31] Mori H., Okazawa H., Iwamoto K., Maeda E., Hashiramoto M., Kasuga M. (2001). A polymorphism in the 5' untranslated region and a Met229-->Leu variant in exon 5 of the human UCP1 gene are associated with susceptibility to type II diabetes mellitus. *Diabetologia*.

[32] Krempler F., Esterbauer H., Weitgasser R. (2002). A functional polymorphism in the promoter of UCP2 enhances obesity risk but reduces type 2 diabetes risk in obese middle-aged humans. *Diabetes*.

[33] Cho Y. M., Ritchie M. D., Moore J. H. (2004). Multifactor-dimensionality reduction shows a two-locus interaction associated with type 2 diabetes mellitus. *Diabetologia*.

[34] D'Adamo M., Perego L., Cardellini M. (2004). The -866A/A genotype in the promoter of the human uncoupling protein 2 gene is associated with insulin resistance and increased risk of type 2 diabetes. *Diabetes*.

[35] Ji Q., Ikegami H., Fujisawa T. (2004). A common polymorphism of uncoupling protein 2 gene is associated with hypertension. *Journal of hypertension*.

[36] Lindholm E., Klannemark M., Agardh E., Groop L., Agardh C. D. (2004). Putative role of polymorphisms in UCP1-3 genes for diabetic nephropathy. *Journal of diabetes and its complications*.

[37] Sasahara M., Nishi M., Kawashima H. (2004). Uncoupling protein 2 promoter polymorphism -866G/A affects its expression in -Cells and modulates clinical profiles of Japanese type 2 diabetic patients. *Diabetes*.

[38] Wang H., Chu W. S., Lu T., Hasstedt S. J., Kern P. A., Elbein S. C. (2004). Uncoupling protein-2 polymorphisms in type 2 diabetes, obesity, and insulin secretion. *American journal of physiology Endocrinology and Metabolism*.

[39] Bulotta A., Ludovico O., Coco A. (2005). The common -866G/A polymorphism in the promoter region of the UCP-2 gene is associated with reduced risk of type 2 diabetes in Caucasians from Italy. *The Journal of clinical endocrinology and metabolism*.

[40] Pinelli M., Giacchetti M., Acquaviva F. (2006). *β*2-adrenergic receptor and UCP3 variants modulate the relationship between age and type 2 diabetes mellitus. *BMC medical genetics*.

[41] Rai E., Sharma S., Koul A., Bhat A. K., Bhanwer A. J., Bamezai R. N. (2007). Interaction between the UCP2-866G/A, mtDNA 10398G/A and PGC1*α* p.Thr394Thr and p.Gly482Ser polymorphisms in type 2 diabetes susceptibility in North Indian population. *Human Genetics*.

[42] Sramkova D., Krejbichova S., Vcelak J. (2007). The UCP1 gene polymorphism A-3826G in relation to DM2 and body composition in Czech population. *Experimental and clinical endocrinology & diabetes: official journal, German Society of Endocrinology [and] German Diabetes Association*.

[43] Lin E., Pei D., Huang Y. J., Hsieh C. H., Wu L. S. (2009). Gene-gene interactions among genetic variants from obesity candidate genes for nonobese and obese populations in type 2 diabetes. *Genetic testing and molecular biomarkers*.

[44] Yang M., Huang Q., Wu J. (2009). Effects ofUCP2 -866 G/AandADRB3 Trp64Argon rosiglitazone response in Chinese patients with type 2 diabetes. *British Journal of Clinical Pharmacology*.

[45] Beitelshees A. L., Finck B. N., Leone T. C. (2010). Interaction between the UCP2 -866 G>A polymorphism, diabetes, and *β*-blocker use among patients with acute coronary syndromes. *Pharmacogenetics and Genomics*.

[46] Heidari J., Akrami S. M., Heshmat R., Amiri P., Fakhrzadeh H., Pajouhi M. (2010). Association study of the -866G/A UCP2 gene promoter polymorphism with type 2 diabetes and obesity in a Tehran population: a case control study. *Archives of Iranian Medicine*.

[47] Vimaleswaran K. S., Radha V., Ghosh S., Majumder P. P., Rao M. R., Mohan V. (2010). A haplotype at theUCP1Gene locus contributes to genetic risk for type 2 diabetes in Asian Indians (CURES-72). *Metabolic syndrome and related disorders*.

[48] Vimaleswaran K. S., Radha V., Ghosh S., Majumder P. P., Sathyanarayana Rao M. R., Mohan V. (2011). Uncoupling protein 2and3Gene polymorphisms and their association with type 2 diabetes in asian indians. *Diabetes technology & therapeutics*.

[49] Xiao F., Zheng X., Cui M. (2011). Telomere dysfunction-related serological markers are associated with type 2 diabetes. *Diabetes care*.

[50] Wang L., Du Z., Liu J., Wu M., Song Y., Jiang R. (2012). Association of UCP3, APN, and TNF-*α* gene polymorphisms with type 2 diabetes in a population of northern Chinese Han patients. *Chemical research in Chinese universities*.

[51] Wang S., Se Y.-M., Liu Z.-Q. (2012). Effect of genetic polymorphism of UCP2-866 G/A on repaglinide response in Chinese patients with type 2 diabetes. *Die Pharmazie*.

[52] Shen Y., Wen Z., Wang N. (2014). Investigation of variants in UCP2 in Chinese type 2 diabetes and diabetic retinopathy. *PloS one*.

[53] Nevzat G., Semih D. (2017). UCP2 866 G/A gene (rs659366) polymorphism associated with diabetes type 2 in Turkish population. *Progress in nutrition*.

[54] Su M., Chen X., Chen Y. (2018). UCP2 and UCP3 variants and gene-environment interaction associated with prediabetes and T2DM in a rural population: a case control study in China. *BMC medical genetics*.

[55] Gomathi P., Samarth A. P., Raj N. (2019). The -866G/A polymorphism in the promoter of the _UCP2_ gene is associated with risk for type 2 diabetes and with decreased insulin levels. *Gene*.

[56] Hou G., Jin Y., Liu M., Wang C., Song G. (2020). _UCP2_ -866G/A Polymorphism is Associated with Prediabetes and Type 2 Diabetes. *Archives of medical research*.

[57] Sharma S., Lalrohlui F., Sharma V. (2020). Candidate gene association study of UCP3 variant rs1800849 with T2D in Mizo population of Northeast India. *International Journal of Diabetes in Developing Countries*.

[58] Esterbauer H., Oberkofler H., Liu Y. M. (1998). Uncoupling protein-1 mRNA expression in obese human subjects: the role of sequence variations at the uncoupling protein-1 gene locus. *Journal of lipid Research*.

[59] Brondani L. A., Assmann T. S., de Souza B. M., Boucas A. P., Canani L. H., Crispim D. (2014). Meta-analysis reveals the association of common variants in the uncoupling protein (UCP) 1-3 genes with body mass index variability. *PloS one*.

[60] de Almeida Brondani L., de Souza B. M., Assmann T. S. (2014). Association of the UCP polymorphisms with susceptibility to obesity: case-control study and meta-analysis. *Molecular biology reports*.

[61] Dalgaard L. T. (2011). Genetic variance in uncoupling protein 2 in relation to obesity, type 2 diabetes, and related metabolic traits: focus on the functional -866G>A promoter variant (rs659366). *Journal of obesity*.

[62] de Souza B. M., Assmann T. S., Kliemann L. M. (2012). The presence of the −866A/55Val/Ins haplotype in the _uncoupling protein 2_ (_UCP2_) gene is associated with decreased _UCP2_ gene expression in human retina. *Experimental eye research*.

[63] Souza B. M., Assmann T. S., Kliemann L. M., Gross J. L., Canani L. H., Crispim D. (2011). The role of uncoupling protein 2 (UCP2) on the development of type 2 diabetes mellitus and its chronic complications. *Arquivos brasileiros de endocrinologia e metabologia*.

[64] Crovesy L., Rosado E. L. (2019). Interaction between genes involved in energy intake regulation and diet in obesity. *Nutrition*.

[65] Hong K. W., Kim S. H., Zhang X., Park S. (2018). Interactions among the variants of insulin-related genes and nutrients increase the risk of type 2 diabetes. *Nutrition research*.

[66] Pravednikova A. E., Shevchenko S. Y., Kerchev V. V. (2020). Association of uncoupling protein (Ucp) gene polymorphisms with cardiometabolic diseases. *Molecular medicine*.

[67] de Luis Roman D. A., Aller R., Izaola Jauregui O. (2010). Relation of −55CT polymorphism of uncoupling protein 3 gene with fat mass and insulin resistance in morbidly obese patients. *Metabolism*.

[68] Salopuro T., Pulkkinen L., Lindström J. (2009). Variation in the UCP2 and UCP3genes associates with abdominal obesity and serum lipids: the Finnish Diabetes Prevention Study. *BMC medical genetics*.

[69] Gotzsche P. C. (2000). Why we need a broad perspective on meta-analysis. *Bmj*.

